# A 46-Year-Old Woman with Unilateral Hearing Loss: A Recent Diagnosis of a Birth Condition

**Published:** 2016-09

**Authors:** Jeanette Sáenz-Piñones, Yolanda García-Hidalgo, Beatriz Arellano-Rodríguez

**Affiliations:** 1 *Department of Otorhinolaryngology, Hospital Puerta de Hierro Majadahonda, Madrid, Spain. *; 2 *Department of Neuroradiology, Hospital Puerta de Hierro Majadahonda, Madrid, Spain. *

Cochlear nerve (CN) aplasia refers to the absence of a visible CN on oblique sagittal magnetic resonance images of the lateral aspect of the inner auditory canal (IAC). Magnetic resonance (MR) is the preferred technique in patients with sensorineural hearing loss and/or vertigo; however, computed tomography (CT) is used to evaluate the IAC or facial nerve canal. Three types of aplasia or hypoplasia can be distinguished (Table 1).

**Table 1:** Classification of malformations

Malformation type 1	Stenotic IAC with absence of VIIIth nerve.
Malformation type 2A	Hypoplasia or aplasia of its cochlear branch associated with other inner ear malformations.
Malformation type 2B	Hypoplasia or aplasia of its cochlear branch associated with normal inner ear .

**Fig 1:**
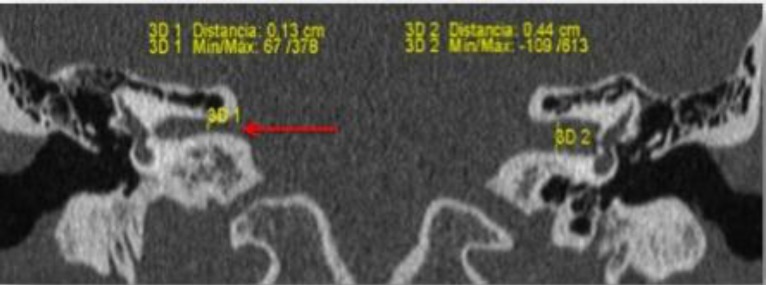
Coronal CT image through the inner ear and inner auditory canal shows a narrow width of right inner auditory canal (red arrow) in comparison to the left

A 46-year-old woman came to our ENT consult after being referred for right hearing loss since childhood. There was neither a history of infections nor tinnitus. Otoscopy was normal. Audiometry showed left normoacusia and right profound sensorineural hearing loss. CT scan showed left middle ear normal, with a focal dehiscence in the tympanic portion of the facial nerve, a right middle ear with the ossicles normal, a focal dehiscence in the tympanic portion of the facial nerve and a reduction in the width of the internal auditory canal with normal anterior and posterior labyrinth (Fig. 1).

**Fig 2:**
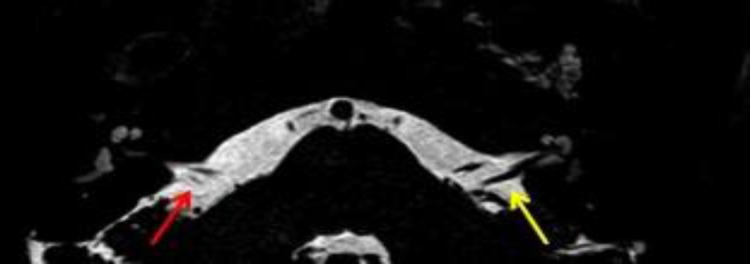
Axial MR 0.6 mm-thick 3D T2 weighted DRIVE shows a narrow width of the eighth cranial nerve (red arrow) in comparison to the left one (yellow arrow) in the cerebellopontine angle

**Fig 3:**
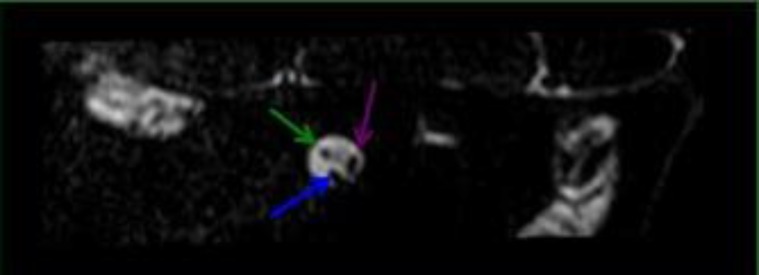
Parasagital reconstruction at the level of the left internal acoustic opening shows the facial nerve (green arrow), cochlear portion (blue arrow) and vestibular portion (purple arrow

MR was performed to thoroughly evaluate the right inner ear, which confirmed hypoplasia of the cochlear nerve (Figs. 2,3).

Cochlear nerve aplasia/hypoplasia can be suspected by CT finding of an internal auditory canal less than 1.5 mm in width because the presence of normal cranial nerves is required for the formation of the IAC and therefore, congenital deficiency of CN VIII (or CN VII) also results in a small or stenotic IAC. It has been reported that IAC stenosis can occur without cochlear nerve hypoplasia; therefore, MR should be performed to detect nerve aplasia or hypoplasia, a challenging diagnosis for the radiologist. In most of the cases, it is associated with profound hearing loss. Nearly all inner ear malformations can be detected on thin-section T2-weighted gradient-echo images or comparable fast spin-echo images. Casselman reported that the cerebellopontine angle should be used as a reference to check the facial and cochlear nerve because he found that the latter was nearly 1 ½-2 times longer than the facial nerve and was never smaller. The nerves are best evaluated and compared on images made perpendicular to the nerves and IAC. This is a case of an adult patient referring to the clinic for right hearing loss since childhood, without ENT evaluation until now. It is important to assess every patient with verified sensorineural hearing loss and no medical history, with radiology tests (CT and MR).

